# SARS-CoV-2 detection using reverse transcription strand invasion based amplification and a portable compact size instrument

**DOI:** 10.1038/s41598-021-01744-y

**Published:** 2021-11-15

**Authors:** Maiken W. Rosenstierne, Shreya Joshi, E. Thomas Danielsen, Helen Webb, Dac Mui Luong, Julie Bjerring, Julie Hindkær, Lærke Jørgensen, Julie Blauenfeldt, Ask Bojesen, Flemming Holck, Johnny Weber Lau, Lars Bangsgaard, Jakob Broberg Lind, Mette Bjergaard Dragheim, Mikkel Rohde Jacobsen, Robert Elkjær, Steven Clauwaert, Kristina Christensen, Charlotta Polacek, Anders Fomsgaard, Tuomas Ojalehto, Antti Tullila, Mirko Brummer, Claus Juel Jensen, Frederikke Holm Jensen, Uffe Vest Schneider, Jan Gorm Lisby, Rikke Lind Jørgensen, Thomas Warthoe, Ebbe Finding, Peter Warthoe

**Affiliations:** 1 Qlife, Borupvang 3, 2750 Ballerup & Symbion, Fruebjergvej 3, 2100 Copenhagen, Denmark; 2 Department of Virus and Microbiological Special Diagnostics, Statens Serum Institut, Artillerivej 5, 2300 Copenhagen, Denmark; 3 Aidian Oy, Espoo, Finland; 4 Klinisk Biokemisk Afdeling, Nordsjællands Hospital, Dyrehavevej 29, 3400 Hillerød, Denmark; 5 Amager og Hvidovre Hospital, Klinisk Mikrobiologisk Afdeling, afsnit 445, Kettegård Allé 30, 2650 Hvidovre, Denmark

**Keywords:** SARS-CoV-2, Viral infection, Integrated optics

## Abstract

Rapid nucleic-acid based tests that can be performed by non-professionals outside laboratory settings could help the containment of the pandemic SARS-CoV-2 virus and may potentially prevent further widespread lockdowns. Here, we present a novel compact portable detection instrument (the Egoo Health System) for extraction-free detection of SARS-CoV-2 using isothermal reverse transcription strand invasion based amplification (RT-SIBA). The SARS-CoV-2 RT-SIBA assay can be performed directly on crude oropharyngeal swabs without nucleic acid extraction with a reaction time of 30 min. The Egoo Health system uses a capsule system, which is automatically sealed tight in the Egoo instrument after applying the sample, resulting in a closed system optimal for molecular isothermal amplification. The performance of the Egoo Health System is comparable to the PCR instrument with an analytical sensitivity of 25 viral RNA copies per SARS-CoV-2 RT-SIBA reaction and a clinical sensitivity and specificity between 87.0–98.4% and 96.6–98.2% respectively.

## Introduction

Severe acute respiratory syndrome coronavirus 2 (SARS-CoV-2), which causes coronavirus disease 2019 (COVID-19), emerged in Wuhan, China in December 2019 and became a worldwide pandemic in March 2020^1^. To this date (15th of June 2021), there has been a total of 175,847,347 confirmed COVID-19 cases and 3,807,276 deaths worldwide (https://covid19.who.int/). The COVID-19 pandemic has become a global crisis, impacting both global health and economy. The return to normality in daily life largely depends on identifying infected individuals, encouraging isolation and quarantine, and development of vaccines against the virus. To this date 2,187,874,534 vaccine doses have been administered (https://covid19.who.int/) but there is still a huge demand for COVID-19 testing and many countries are screening their population for the presence of the virus using Reverse Transcriptase Polymerase Chain Reaction (RT-PCR) and antigen tests^2–4^. RT-PCR is the gold standard nucleic acids amplification test (NAAT) for diagnosis of COVID-19 and many other viral infections due to the high sensitivity and specificity of the method. However, the method is labour intensive, time-consuming (typically > 2 h), and requires transport to laboratories with specialized laboratory equipment and personnel, which can prolong the time to result up to 24 h or longer. This extended turnaround time has pushed the development of simplified NAAT tests that can be performed locally. Simplified RT-PCR workflows has been developed using nucleic acid (NA) extraction free methods such as direct lysis or heat^5–9^ or alternative NAAT tests such as loop-mediated isothermal amplification (RT-LAMP)^10–17^, CRISPR-Cas12 and CRISPR-Cas13a based systems^18,19^, recombinase polymerase amplification (RT-RPA)^20–22^, and nicking-endonuclease amplification reactions (RT-NEAR)^23,24^ which can reduce the overall reaction time to approximately 20–30 min. Some of these NAAT tests has been developed to be performed outside specialized laboratories such as the Cue COVID-2 test^25^, the Visby medical SARS-CoV-2 test^26^ and the DNAnudge Covid-19 test^27^.

Here we present a new compact size portable point of care (POC) system called the Egoo Health System, which consists of an Egoo instrument (470 g), an Egoo clinical application (app), and an Egoo assay capsule. The Egoo Health System was initially designed for detection of biomarkers in blood, saliva and urine, but here we show that the Egoo Health System can also be used to detect SARS-CoV-2 from oropharyngeal swabs. The compact size Egoo instrument is designed for use with multiple assay types, each utilizing a single-use, test specific assay capsule, thus the Egoo Health System can be used for several different assays, including both biochemical and NAAT tests. After an assay run, raw data is sent via WiFi to the Egoo server for raw data analysis, and the calculated assay result is sent to a laptop/smartphone, which is the primary display for the Egoo Health System. The Egoo Health System was initially developed for use in private homes but can also be used in primary care clinics, nursing homes, and workplaces, without the need of specialized laboratory staff.

For detection of SARS-CoV-2 with the Egoo Health system we developed an SARS-CoV-2 Egoo capsule based on the previously described reverse transcription strand invasion based amplification (RT-SIBA) isothermal technology and SYBR green detection^28–31^. The SARS-CoV-2 RT-SIBA assay can be performed directly on crude oropharyngeal swabs without NA extraction with a reaction time of 30 min.

## Methods

### Ethical statement

The study is not a medical health science research project but a method comparison study using surplus material from routine oropharyngeal samples collected at Nordsjællands hospital and Amager and Hvidovre University hospital. The need for informed consent and ethical approval were reviewed by the Institutional Review Board at Amager and Hvidovre University Hospital and Nordsjællands hospital and the regional research ethic committee for Region Hovedstaden (The National Committee on Health Research Ethics, Region Hovedstaden, Blegdamsvej 60, 1. Sal opgang 94A11, DK-2100 Copenhagen) and found not to need approval according to national ethic research regulation.

### The Egoo Health System

The Egoo Health System comprises of an Egoo instrument (capsule reader), an Egoo power adaptor, an Egoo clinical application (app), and an Egoo assay capsule. The Egoo instrument is compact in size (W66 × H107 × D94 mm), and weight (470 g/1.04 lbs). It consists of an integrated optical microelectromechanical system (optical MOEM)^32^ and has a dual optical unit for simultaneously measuring fluorescence and absorbance. The optical unit is integrated on a micro heating (max. 50 °C (122°F)) and vortex mixing unit (max. 3000 rpm) for heating and mixing the assay reagents during assay runs. The Egoo instrument has a mechanical piston mechanism which via a plunger unit can tightly seal the Egoo assay capsule after applying the sample, resulting in a closed system. In addition, the piston mechanism can inject various reagents from the integrated capsule injection chambers which sit on top of each assay specific Egoo capsule. Instrument specifications can be seen in supplementary Table S1. The SARS-CoV-2 Egoo capsule (W24 × H28 × D36 mm) (supplementary Table S2) consists of 140 µl frozen SARS-CoV-2 RT-SIBA Mastermix. The SARS-CoV-2 Egoo capsules are stored at − 20 °C until their intended use. Stability studies of the SARS-CoV-2 Egoo capsules are ongoing, but the current shelf-life of the SARS-CoV-2 Egoo capsule is 180 days at  − 20 °C (supplementary Fig. S1).

### Egoo data analysis

After each assay run, the raw data is sent via WiFi to the Egoo server for raw data analysis. For the SARS-CoV-2 RT-SIBA assay, the calculations are based on 3 fluorescence measurements recorded every minute for 30 min. The fluorescence measurements are graphed via an algorithm within the server. Based on an initial data set of > 100 positive/negative oropharyngeal patient samples used to define minimum and maximum values within the algorithm (data not shown), the assay result is determined. The primary display for the Egoo Health System is a laptop/smartphone. Based on the accumulated curve and slope, the assay result is reported as either “negative”, “positive” or “inconclusive” to the end user. Alternatively, the raw data can be analysed via Excel or other graphing programs, should the end user wish to visualize the resulting amplification curve from the reaction.

### Sample preparations and dilutions

Oropharyngeal swabs collected with flocked swabs (Jiangsu Hanheng Medical Technology, Copan) from patients were dissolved directly into either 1 ml SIBA lysis/reaction buffer (Aidian/Qlife), 1 ml PBS (Gibco), 1 ml Universal Transport Media (UTM) (Copan), 1 ml Virus Transport Media (VTM) (Nest Biotechnology) or 1 ml VTM (Mole Bioscience). To release virus from the flocked swabs and the sample collection tubes were incubated for 10 min at room temperature. Samples dissolved in PBS, UTM, VTM or antigen buffers (GenSure COVID-19 Antigen Rapid Test, Acro 2019-nCoV IgG/IgM Rapid Test, Biosynex Covid-19 Ag BSS Rapid Test, SD Biosensor COVID-19 Ag Test were diluted 10-fold in SIBA lysis/reaction buffer (containing mild detergents and 80 mM Mg-acetate) before being applied to the SARS-CoV-2 RT-SIBA Mastermix (Qlife). Samples undergoing SARS-CoV-2 testing by RT-PCR on the Corbas Liat System at Nordsjællands Hospital were used without further preparation according to the manufactures instructions. Samples undergoing SARS-CoV-2 testing by RT-PCR on the BGI system at Hvidovre Hospital were total NA purified on a MGISP-960 using the MGIEasy Magnetic Beads Virus DNA/RNA Extraction Kit (MGI Tech Co., Ltd.). The sample extraction amount was 180 µl and total NA was eluted in 33 µl.

### SARS-CoV-2 RT-SIBA

The SARS-CoV-2 RT-SIBA assay consist of a forward primer (5′-GAACTTTAAGTCAGTTCTT-3′), reverse primer (5′-CAGTCTCAGTCCAACA-3′) and an invasion oligo containing a Cytosine 5′ overhang and 2′-O-methyl RNA moieties in the 3′end (5′- CCCCCCCCCCCCCCTTTATTATCAAAACAATGTTTTTATGTCTG*AAGCAAAATGTT*-3′). The 2′-O-methyl RNA moieties are shown in italic letters. The final SARS-CoV-2 Mastermix (Qlife) consists of mixing three reagents according to the following protocol. The RT-SIBA Mix A (Aidian), Mix B (Aidian) and Oligomix (Aidian) were thawed on ice and mixed by vortexing. Mix A can in some instances form precipitates which can be re-dissolved by heating to 37–41 °C followed by vortexing. For SARS-CoV-2 RT-SIBA reactions performed in a PCR instrument the mastermix was prepared by mixing 7 µl Mix A, 7 µl Mix B, and 3.5 µl Oligomix per reaction. Mastermix (17.5 µl) was added to PCR tubes and 2.5 µl of sample (diluted 10-fold in SIBA lysis/reaction buffer) was added to the mastermix. The RT-SIBA reactions were performed using either the MX3005P (Stratagene) or CFX96 (Bio-Rad) PCR instrument. Fluorescence measurements were recorded every minute for 30 min at 44 °C, followed by a melt curve analysis: 44–95 °C. For reactions performed in the Egoo instrument, SARS-CoV-2 Egoo capsules (Qlife) were prepared by adding 140 µl of premade mastermix to the capsule cuvette following final sealing and assembly of the Egoo capsule. The SARS-CoV-2 Egoo capsules can be stored at − 20 °C for more than 180 days without any influence on the time to positive (supplementary Fig. S1). Upon use the SARS-CoV-2 Egoo capsules are thawed before being applied with 20 µl of sample (diluted 10-fold in SIBA lysis/reaction buffer). The in-use stability of the SARS-CoV-2 capsule after thawing is 8–24 h if kept at 30–20 °C respectively (supplementary Fig. S2). After application of the sample to the SARS-CoV-2 Egoo capsule the capsule must be loaded into the Egoo instrument within 15–30 min (supplementary Fig. S3). Before the reaction starts, the Egoo capsule is closed in the Egoo instrument once the piston mechanism seals the capsule with the plunger. Once the plunger has sealed the capsule tight, the Egoo instrument heats the capsule to 44 °C for 30 min. During the reaction in the Egoo instrument, the reagents within the capsule are mixed by vortexing (1000 rpm) for 3 s every 5 min. Fluorescence measurements are recorded 3 times every minute.

### SARS-CoV-2 RT-PCR

The E-gene assay from Charité Berlin^33^ was used as a single plex RT-PCR assay at Qlife with the Luna Universal Probe One-Step RT-qPCR Kit (New England Biolabs). Briefly described 12.5 µl Luna Universal One-Step Reaction Mix, 1.25 µl Luna WarmStart RT Enzyme Mix, 0.5 µl E_Sarbeco_F1 (20 µM), 0.5 µl E_Sarbeco_R2 (20 µM), 0.25 µl E_Sarbeco_P1 (20 µM), 7.5 µl nuclease-free water and 2.5 µl sample (purified RNA or sample diluted 10-fold in SIBA lysis/reaction buffer) were mixed and run with the following program: 10 min at 55 °C, 3 min at 95 °C, 45 cycles of 15 s. at 95 °C and 30 s. at 58 °C. The RT-PCR reactions were performed using either the MX3005P (Strategene), CFX96 (Bio-Rad), or AriaMx (Agilent), and fluorescence were captured using the FAM channel after each cycle. At Nordsjællands hospital the Cobas SARS-CoV-2 & Influenza A/B NAAT test on the Cobas Liat System (Roche) was used directly (200 µl) in the cartridge according to the manufactures instructions. At Hvidovre Hospital a newly laboratory developed multiplex test adopted from^34^ targeting the E-gene (Charité Berlin)^33^, the N2-gene in SARS-CoV-2 and RNaseP as human target was used for RT-PCR analysis in the BGI system. Reactions were set up in a 20 µl reaction volume using 8 μL sample (purified RNA), 10 µl KiCqStart One-Step Probe RT-qPCR ReadyMix (Sigma-Aldrich), 1 µl 4 mM dUTP (0.2 mM in total) and 1 µl mix of primers and probes. Final concentrations of primers and probes were 500 nM CoV_E_F primer (5′-ACAGGTACGTTAATAGTTAATAGCGT-3′), 400 nM CoV_E_R primer (5′-ATATTGCAGCAGTACGCACACA-3′), 150 nM CoV_E_P probe (5′-LC610-ACACTAGCCATCCTTACTGCGCTTCG-BBQ-3′), 400 nM CoV_N2_F primer (5′-TTACAAACATTGGCCGCAAA-3′), 400 nM CoV_N2_R1 primer (5′-AAGGTGTGACTTCCATGCCA-3′), 150 nM CoV_N2_P FAM probe (5′-FAM-ACAATTTGCCCCCAGCGCTTCAG-BBQ-3′), 100 nM RNaseP_F primer (5′-AGATTTGGACCTGCGAGCG-3′), 100 nM RNaseP_R primer (5′-GAGCGGCTGTCTCCACAAGT-3′) and 125 nM RNaseP_P_Cy5 probe (5′-Cy5-TTCTGACCTGAAGGCTCTGCGCG-BBQ-3′). RT-PCR was performed on the LineGene 9600 instrument with the following PCR profile: 10 min of 50 °C and 60 s of 95 °C followed by 45 cycles of 95 °C for 5 s and 60 °C for 30 s. The fluorescence was captured using the FAM, Cy5 and ROX channels after each cycle.

### Virus culture and RNA purification

Inactivated virus cultures for Epstein-Barr Virus (EBV)(B95-8), Parainfluenza virus type 1 (PIV-1), Adenovirus type 5 (Adv5), Respiratory Syncytial virus type A (RSV-A)(2006), Influenza A (H1N1pdm), (INFL A)(NY/02/07), Influenza A (H3N2), Influenza B (INFL B)(Yamagata/16/88), Rhinovirus A16, Enterovirus type 68 (EV-68)(2007), Human metapneumovirus (hMPV) (Peru2-2002), Coronavirus OC43, Coronavirs NL63, Coronavirus 229E, SARS-COV-2 (Italy-INMI1) (1.02 × 10^8^ TCID_50_/mL), SARS-COV-2 (USA-WA1/2020) (3.09 × 10^8^ TCID_50_/ml), SARS-COV-2 (Hong Kong/VM2000i06i/2020)(1.15 × 10^7^ TCID_50_/mL) purchased from Helvetica Health Care were used directly by spiking into an oropharyngeal swab background resulting in a 10-fold dilution of the virus. QCMD panels for MERS (2019), RSV (2019), hMPV (2019) and coronavirus (2019) were purified using the MagNA Pure 96 system (Roche) and the DNA and Viral NA Small Volume Kit (Roche). The human SARS-CoV-2 isolate 2019-nCoV Munchen 1-2 2020/984 (026V-03883, EVAg) was cultured in VERO E6 cells, and the virus titre of the supernatant was determined to 1.6 × 10^7^ TCID_50_/ml. In addition, the harvested supernatant was quantified to 1.2 × 10^7^ RNA copies/ml using MagNA Pure purified RNA and a standard curve based on the synthetic SARS-CoV-2 RNA control (MT007544.1, Twist Bioscience) spiked into RNA from a SARS-CoV-2 negative oropharyngeal swab. The quantification was performed using the RT-PCR E-gene assay^33^.

### Clinical samples

Retrospective SARS-CoV-2 positive and negative oropharyngeal patient samples were used to analyse the clinical sensitivity of the SARS-CoV-2 Egoo capsule using the Egoo instrument. 227 oropharyngeal swabs dissolved in PBS and diagnosed positive or negative for SARS-CoV-2 using direct lysis and the E-gene RT-PCR assay^33^ from the Qlife COVID-19 Service Center were analyzed. Informed patient consent was obtained for Qlife patient samples. In addition, two independent method comparison studies were performed at two different hospitals. At Hvidovre and Amager hospitals, 700 retrospective oropharyngeal swabs dissolved in UTM previously diagnosed positive or negative for SARS-CoV-2 using the SARS-CoV-2 Roche Flow/MGI-BGI RT-PCR assay were re-tested followed by analysis using the SARS-CoV-2 Egoo capsule on the Egoo instrument. At Nordsjællands hospital, 224 patient samples diagnosed positive or negative for SARS-CoV-2 using the SARS-CoV-2 Cobas Liat System (Roche) were re-tested within 24 h using the SARS-CoV-2 Egoo capsule on the Egoo instrument. All oropharyngeal patient swabs were collected in accordance with national guidelines and regulations and only surplus material from routine oropharyngeal samples were used in this study.

## Results

### The Egoo Health System

**Figure 1:**
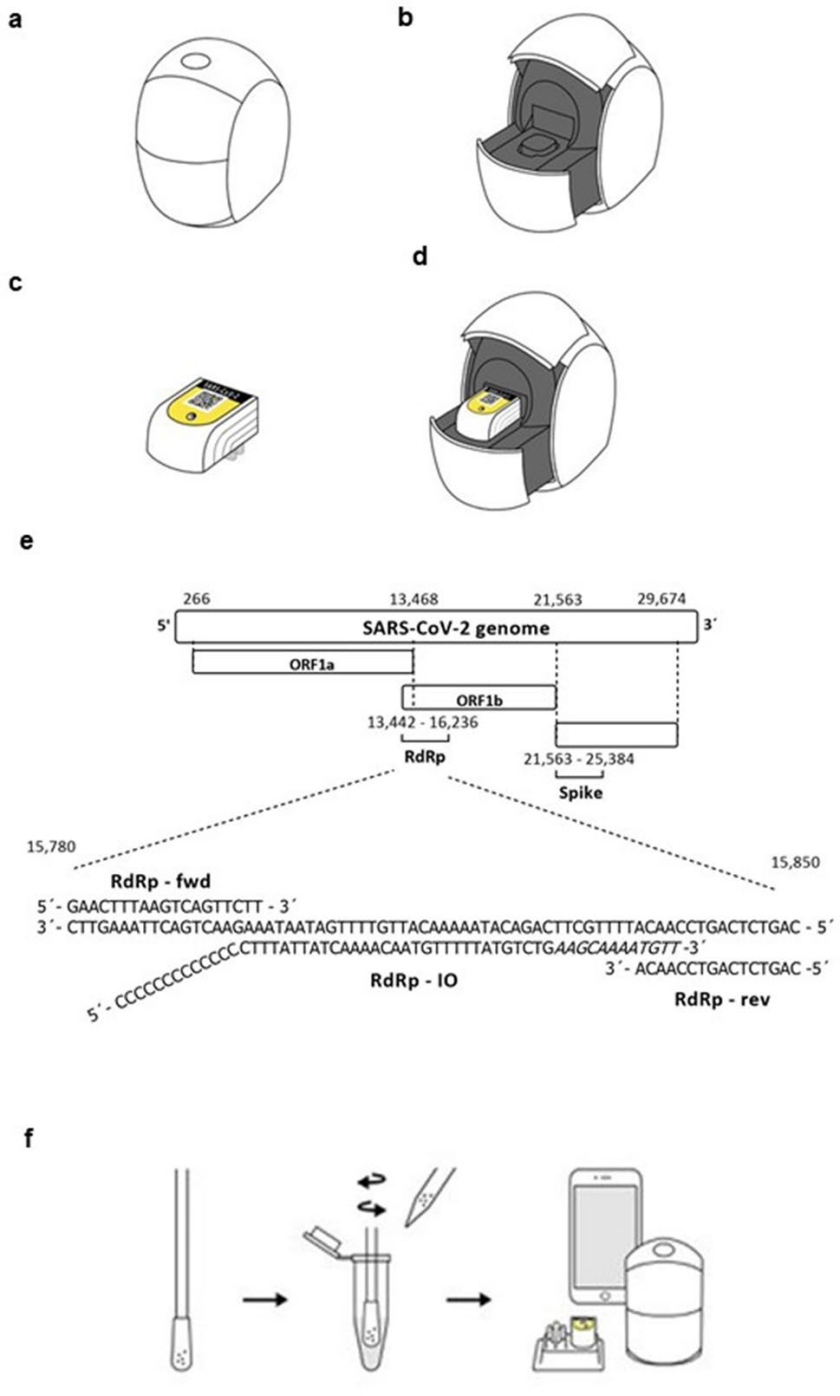
The Egoo Health System and the SARS-CoV-2 RT-SIBA assay. (**a**) The Egoo instrument in the closed position. (**b**) The Egoo instrument in the open position. (**c**) The SARS-CoV-2 Egoo capsule. (**d**) The SARS-CoV-2 Egoo capsule placed into the Egoo instrument. (**e**) The SARS-CoV-2 genome with the RdRp gene and the position of the SARS-CoV-2 RT-SIBA assay is shown. The sequence of the invasion oligo (RdRp IO) with the Cytosine (C) overhang and 2′-O-methyl RNA (Italic letters) and the forward (RdRp-fwd) and reverse (RdRp-rev) primers are shown. (**f**) The sampling workflow for the SARS-CoV-2 Egoo Health system. The swab is directly dissolved in SIBA lysis/reaction buffer and 20 µl of the sample is transferred to the SARS-CoV-2 Egoo capsule and analysed in the Egoo instrument.

The Egoo Health System consists of a small Egoo instrument, a laptop or mobile phone, with the Egoo clinical app, and an Egoo capsule containing the assay of interest (Fig. 1a–d). Because of the limited heating system in the Egoo instrument (max. 50 °C), we developed an isothermal SARS-CoV-2 RT-SIBA assay for the Egoo capsule that target the RdRp gene in SARS-CoV-2 genome (Fig. 1e). In silico analysis of 699,737 full-length SARS-CoV-2 sequences submitted to GISAID^35^ in the period from 26th of December 2019 to 22nd of May 2021 show that 99.63% of all worldwide isolates would be detected by the SARS-CoV-2 RT-SIBA assay including the UK (B.1.1.7), South Africa (B.1.351), Brazil (P.1) and Indian (B.1.1617.2) variants (Supplementary Table S3). Only 0.37% of the sequences analysed contained SNPs in either the binding region of the primers or the invasion oligo. A representative figure of the observed SNPs can be seen in supplementary Fig. S4. For methodological simplification of the Egoo Health system, the SARS-CoV-2 RT-SIBA assay was designed to be used directly on crude samples without NA extraction using a SIBA lysis/reaction buffer (Fig. 1f), which contain mild detergents and magnesium for activation of the SARS-CoV-2 RT-SIBA assay.

### The analytical specificity of the SARS-CoV-2 RT-SIBA assay

First, we wanted to investigate the analytical specificity of the SARS-CoV-2 RT-SIBA assay against other human coronaviruses (hCoV-NL63, hCoV-229E and hCoV-OC43) and the most common human respiratory viruses such as Influenza A H1N1, Influenza B and Respiratory Syncytial virus (RSV) (Table 3). Purified viral NA or inactivated virus cultures from the different viruses were spiked into a SARS-CoV-2 negative oropharyngeal background. The samples were diluted 10-fold in SIBA lysis/reaction buffer before being analysed with the SARS-CoV-2 RT-SIBA assay using two different PCR instruments (MX3005P or CFX96) and the small Egoo instrument using SARS-CoV-2 Egoo capsules (Table 1). Additional specificity testing of the SARS-CoV-2 Egoo capsules against a wide range of common human viruses and bacteria can be seen in supplementary Table S4. No cross-reactivity to other respiratory viruses or bacteria was observed for either the purified NA or non-purified viral and bacterial cultures and the SARS-CoV-2 RT-SIBA assay was 100% specific for SARS-CoV-2.

**Table 1:** Analytical specificity of the SARS-CoV-2 RT SIBA assay.

Sample material	Virus content	Number of positives, PCR instrument	Number of positives, Egoo instrument
Purified NA	hCoV-NL63	0/4*	na
	hCoV-OC43	0/3*	na
	hCoV-229E	0/1*	na
	hCoV-HKU1	0/1*	na
	MERS	0/5*	na
Virus culture spiked into oropharyngeal swab (non-purified NA)	hCoV-NL63	0/5^#^	0/5
	hCoV-229E	0/5^#^	0/5
	RSV	0/8^#^	0/5
	hMPV	0/8^#^	0/5
	EBV	0/5^2#^	0/5
	PIV-1	0/5^#^	0/5
	ADV5	0/5^#^	0/5
	INFL A H1N1pdm	0/5^#^	0/5
	INFL B	0/5^#^	0/5
	Enterovirus 68	0/5^#^	0/5
	SARS-CoV-2	5/5^#^	5/5

NA, nucleic acids; INFL, Influenza virus; pdm, 2009 pandemic; RSV, Respiratory Syncytial Virus; hCoV, Human Corona virus; MERS, Middel East Respiratory syndrome related coronavirus; hMPV, Human Metapneumovirus; EBV, Epstein Barr virus, PIV-1, Parainfluenza virus type 1; ADV5, adenovirus type 5; na: not analyzed.

*MX3005P (Strategene).

^#^CFX96dx (Bio-Rad).

### The analytical sensitivity of the SARS-CoV-2 RT-SIBA assay

**Figure 2:**
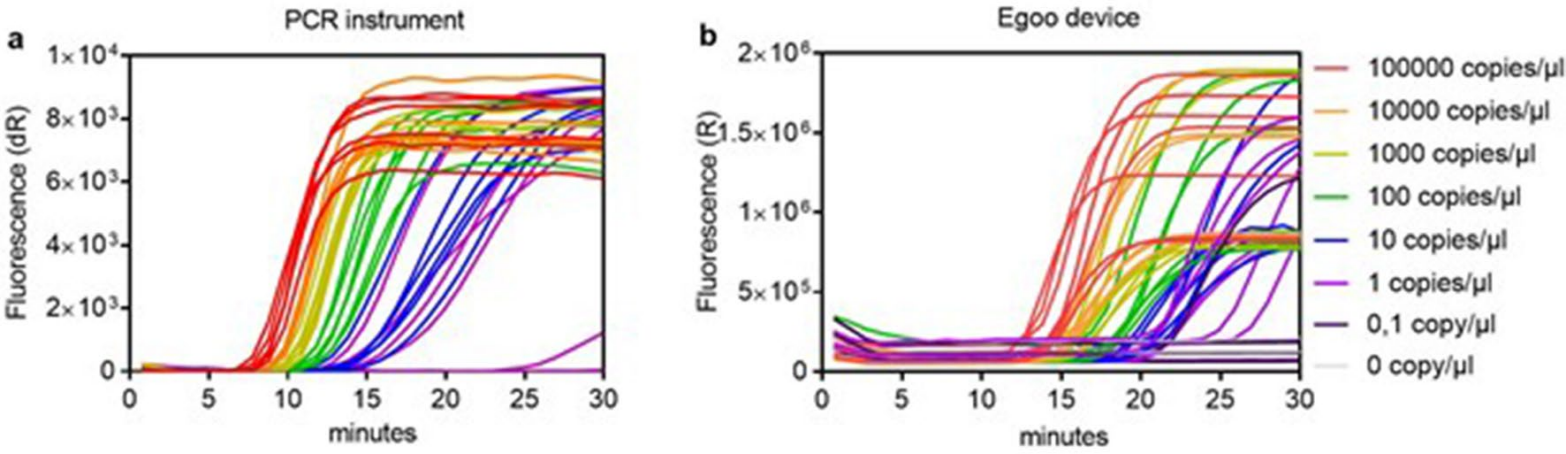
The analytical sensitivity of the SARS-CoV-2 RT-SIBA assay. Amplification curves of synthetic SARS-CoV-2 RNA diluted in negative oropharyngeal swab dissolved in SIBA lysis/reaction buffer and analysed in the (**a**) MX3005P PCR instrument (n = 6) or in (**b**) five different non-calibrated Egoo instruments (n = 6). The different concentrations (RNA copies/µl) of synthetic RNA are color coated on both graphs.

Next, we wanted to test the analytical sensitivity of the SARS-CoV-2 RT-SIBA assay. Synthetic SARS-CoV-2 RNA and inactivated SARS-CoV-2 virus culture was spiked into negative oropharyngeal swab at different concentrations and diluted 10-fold in SIBA lysis/reaction buffer. The different dilutions were tested using both a PCR instrument (Mx3005P) and five different Egoo instruments (Fig. 2a,b, Table 2). Figure 2 shows the amplification curves of the synthetic SARS-CoV-2 RNA from the PCR instrument (Fig. 2a) and five different non-calibrated Egoo instruments (Fig. 2b). For the PCR instrument, the dilutions were predominantly detected with 8–16 min, whereas the dilutions were detected within 12–22 min on the different Egoo instruments (Fig. 2, Table 2). For the endpoint fluorescence signal, differences between the Egoo instruments were observed which indicated that the Egoo instruments were not calibrated, however, this did not influence the calculated results from the backend server. The limit of detection (LOD) of the SARS-CoV-2 RT-SIBA assay was found to be between 20 and 25 RNA copies/reaction when using both the synthetic RNA and whole virus culture. No difference between the LOD was observed between the PCR instrument and the five Egoo instruments tested (Table 2). However, for the PCR instrument only 2.5 µl of sample was loaded into the PCR tube containing 17.5 µl SARS-CoV-2 RT-SIBA mastermix, whereas 20 µl of sample was loaded into the SARS-CoV-2 Egoo capsule (containing 140 µl of mastermix). This corresponds to a lower sample input concentration for the Egoo instrument compared to the PCR instrument. The SARS-CoV-2 Egoo Health system can detect as low as 1.3 viral RNA copies/µl (Table 2).

**Table 2:** Analytical sensitivity of the SARS-CoV-2 assay.

Sample material	SARS-CoV-2 RT-SIBA on PCR instrument^a^				SARS-CoV-2 RT-SIBA on Egoo instrument			
	Viral load of sample (cp/µl)	Copies to RT-SIBA reaction* (cp)	No. of Pos. (%)	Min. to pos (mean ± SD)	Viral load of sample (cp/µl)	Copies to RT-SIBA reaction^¤^ (cp)	No. of Pos. (%)	Min. to pos (mean ± SD)
Synthetic RNA^b^	1.0 × 10^5^	2.5 × 10^5^	6/6 (100)	8.5 ± 0.5	1.0 × 10^5^	2.0 × 10^6^	8/8 (100)	16.0 ± 1.5
	1.0 × 10^4^	2.5 × 10^4^	6/6 (100)	9.6 ± 0.1	1.0 × 10^4^	2.0 × 10^5^	6/6 (100)	16.7 ± 2.1
	1.0 × 10^3^	2.5 × 10^3^	6/6 (100)	10.3 ± 0.4	1.0 × 10^3^	2.0 × 10^4^	6/6 (100)	17.2 ± 1.7
	1.0 × 10^2^	2.5 × 10^2^	6/6 (100)	11.7 ± 0.5	1.0 × 10^2^	2.0 × 10^3^	6/6 (100)	19.8 ± 2.3
	**1.0 × 10**^**1**^	**2.5 × 10**^**1**^	**6/6 (100)**	**14.3 ± 1.1**	1.0 × 10^1^	2.0 × 10^2^	6/6 (100)	21.5 ± 3.3
	1.0 × 10^0^	2.5 × 10^0^	5/6 (83)	16.9 ± 0.9	**1.0 × 10**^**0**^	**2.0 × 10**^**1**^	**7/7 (100)**	**25.6 ± 3.3**
	1.0 × 10^–1^	2.5 × 10^–1^	na	na	1.0 × 10^–1^	2.0 × 10^0^	1/8 (13)	22.9
	0	0	0/6	0	0	0	0/6	0
Virus culture^c^	1.2 × 10^3^	2.9 × 10^3^	64/64 (100)	12.6 ± 1.1	1.2 × 10^3^	2.4 × 10^3^	2/2 (100)	16.3 ± 0.7
	1.2 × 10^2^	2.9 × 10^2^	64/64 (100)	15.9 ± 1.9	1.2 × 10^2^	2.4 × 10^2^	1/1 (100)	21.88
	1.0 × 10^1^	2.6 × 10^1^	33/33 (100)	20.8 ± 3.0	1.2 × 10^1^	1.0 × 10^2^	2/2 (100)	21.4 ± 0.7
	**9.3 × 10**^**0**^	**2.3 × 10**^**1**^	**32/33 (97)**	**19.9 ± 2.2**	6 × 10^0^	5.0 × 10^1^	2/2 (100)	21.9 ± 1.4
	**8.1 × 10**^**0**^	**2.0 × 10**^**1**^	**33/33 (100)**	**20.1 ± 1.8**	**1.3 × 10**^**0**^	**2.5 × 10**^**1**^	**19/20 (95)**	**21.8 ± 1.8**
	6.3 × 10^0^	1.6 × 10^1^	31/33 (94)	21.9 ± 3.9	1 × 10^0^	2.0 × 10^1^	9/20 (45)	23.9 ± 2.9
	0	0	0/33	0	0	0	0/6	0

The bold highlights define the limit of detection.

na, not analyzed; no, number; Pos, positive; min, minutes; SD, standard deviation.

^a^MX3005P.

^b^Twistsyntetic SARS-COV-2 RNA ctr1.

^c^2019-nCoV isolate (026V-03883) (EVAg).

*2.5 µl sample input to SARS-CoV-2 RT-SIBA reaction when the RT-SIBA reaction is performed in a PCR instrument, final volume 20 µl.

^¤^20 µl sample input to SARS-CoV-2 Egoo capsule, when the RT-SIBA reaction is performed in the Egoo instrument, final volume 160 µl.

### Extraction free sample handling

**Figure 3:**
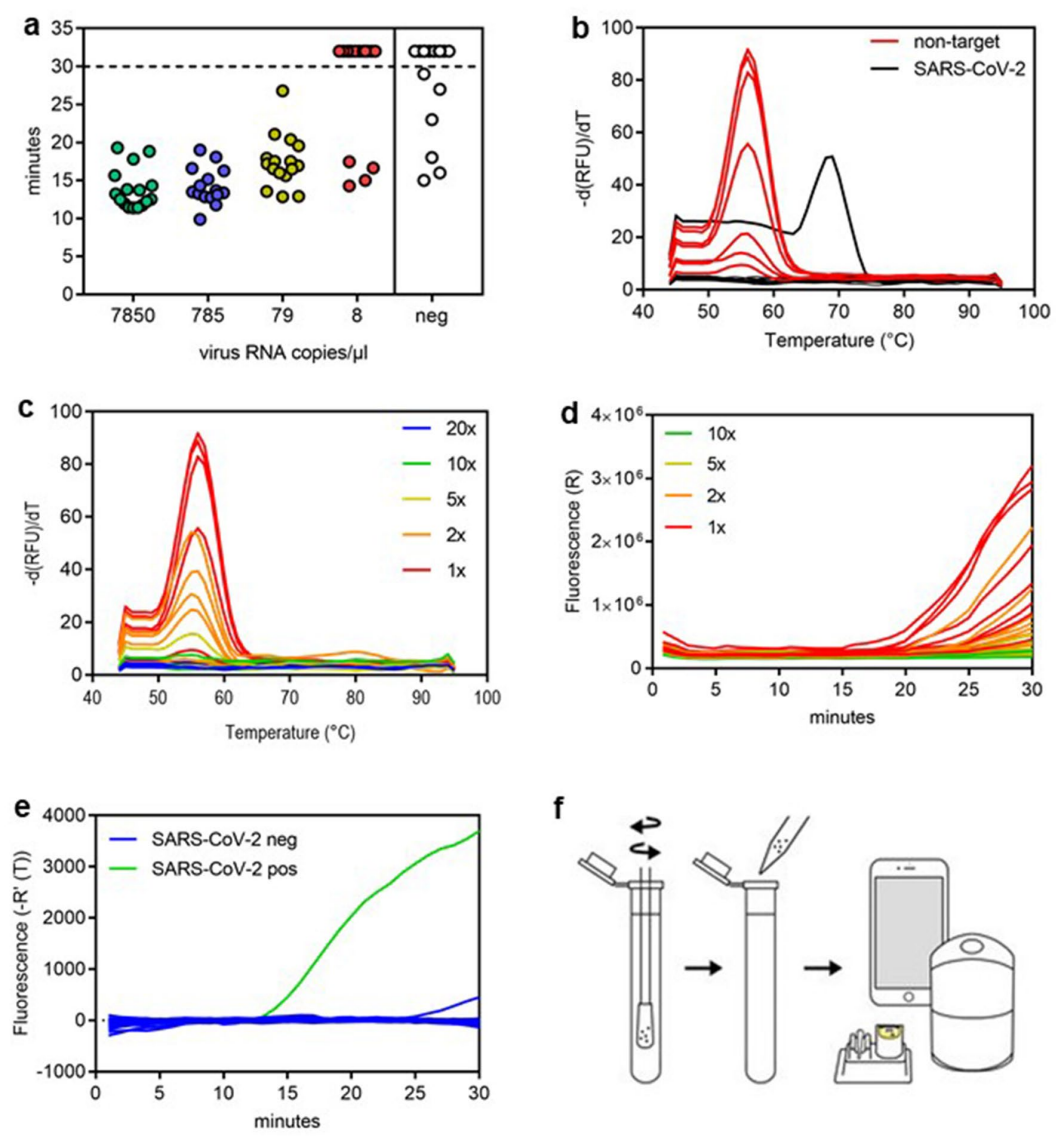
The sampling method for the SARS-CoV-2 RT-SIBA assay. (**a**) Analysis of SARS-CoV-2 spiked swabs dissolved directly in SIBA lysis/reaction buffer and analysed with the SARS-CoV-2 RT-SIBA assay in a PCR instrument (MX3005P). The graph shows time to positive in minutes and the calculated hypothetical virus concentration (virus RNA copies/µl) of the sample after the swab has been dissolved in 500 µl SIBA lysis/reaction buffer (n = 16). For graphical appearance samples that are not detected are given the hypothetical value of 32 min which is shown above the final reaction time (30 min) (dotted line). (**b**) Melting curve analysis of the false positive (non-target) signal detected in the negative oropharyngeal swabs. The melting peak for a true positive signal (black) is shown (68 °C) and the melting peak for the false positives (non-target) (red) (56 °C) is shown. (**c**) Melting peaks from different dilutions of the false-positive samples. The melting peak from the different dilutions (1×, 2×, 5×, 10×, and 20×) are color coated. (**d**) Analysis of false positive samples in the Egoo instrument. The amplification curves from the different dilutions (1×, 2×, 5×, and 10×) are color coated. (**e**) Analysis of negative oropharyngeal swabs diluted 10-fold in SIBA lysis/reaction buffer (n = 128). Amplification curves from the SARS-CoV-2 positive control (green) and the negative oropharyngeal swabs (blue) are shown. (**f**) The sampling workflow for the SARS-CoV-2 Egoo Health system. The swab is directly dissolved in 10 ml SIBA lysis/reaction buffer and 20 µl is transferred to the SARS-CoV-2 Egoo capsule and analysed in the Egoo instrument.

The SARS-CoV-2 RT-SIBA assay was designed to be used directly on crude samples without NA extraction using a SIBA lysis/reaction buffer (Fig. 1f). To test if lysis can be performed directly in the sample collection tube, different concentrations of SARS-CoV-2 virus culture were spiked into a negative oropharyngeal swab background and subsequently added to sampling swabs (n = 16). SIBA lysis/reaction buffer (500 µl) was added directly to the tubes containing the spiked swabs and the tubes were incubated for 10 min at RT with shaking to release the viral RNA from the swab, before being analysed by the SARS-CoV-2 RT-SIBA assay using a PCR instrument. As negative controls, negative oropharyngeal swabs (n = 18) directly dissolved SIBA lysis/reaction buffer were also analysed (Fig. 3a). Swabs spiked with 4200 viral RNA copies and dissolved in SIBA lysis/reaction buffer to a concentration of 79 viral RNA copies could easily be detected with the SARS-CoV-2 RT-SIBA reaction (Fig. 3a). Analysis of SARS-CoV-2 negative oropharyngeal swabs showed positive detection in 39% (7/18) of the samples (Fig. 3a) and analysis of the melting curve showed a second melting peak around 56 °C, whereas the correct melting peak for a SARS-CoV-2 positive sample should be at 68 °C (Fig. 3b). The SARS-CoV-2 assay is based on a SYBR Green detection of the amplified product, and therefore any non-target amplification occurring due to the genomic background in the sample is also detected. This non-target amplification is easily distinguishable from target-specific amplification in a PCR instrument by performing melt curve analysis. Since melt curve analysis cannot be performed in the Egoo instrument, we investigated if diluting the samples by 2-, 5-, 10-, 20-fold would reduce non-target amplification (Fig. 3c,d). Dilution of the samples clearly showed that 10- and 20-fold dilutions of the samples eliminated the formation of the non-target melting peak (Fig. 3c,d). To further test this, 128 negative oropharyngeal patient swabs (dissolved in 1 ml PBS) were 10-fold diluted in SIBA lysis/reaction buffer and analysed with the SARS-CoV-2 RT-SIBA assay. Only 1/128 (0.7%) of the 10-fold diluted negative oropharyngeal swabs showed non-target amplification, with a very small amplification curve (Fig. 3e). These results show that samples must be diluted 10-fold in SIBA lysis/reaction buffer before being analysed on the Egoo instrument. To methodologically simplify the dilution procedure for non-professionals, 10 ml of SIBA lysis/reaction buffer must be added to a sample collection tube if the sample is to be used directly with the SARS-CoV-2 Egoo capsule and the Egoo instrument (Fig. 3f).

### Compatibility to sample media

**Figure 4:**
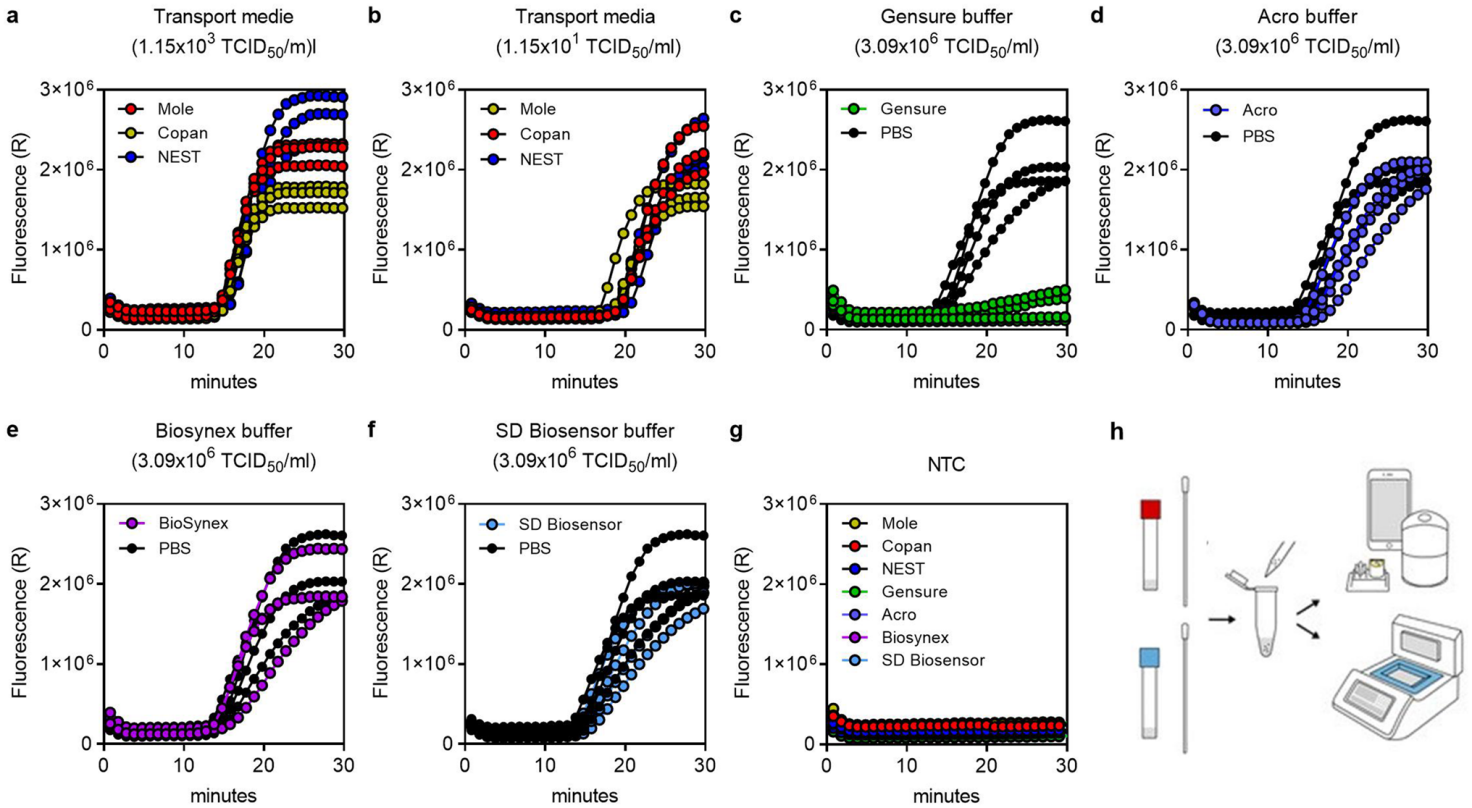
Analysis of the SARS-CoV-2 Egoo Health System using different sampling medium. (**a**, **b**) Amplification curves for two dilutions of the SARS-CoV-2 virus isolate (Hong Kong VM20001061/2020) in different transport medium (UTM (Copan), VTM (Mole Bioscience and Nest Biotechnology) (n = 3). (**c**–**f**) Amplification curves for the SARS-CoV-2 virus isolate (USA-WA1/2020) dissolved in different antigen test sampling medium (GenSure COVID-19 Antigen Rapid Test, Acro 2019-nCoV IgG/IgM Rapid Test, Biosynex Covid-19 Ag BSS Rapid Test, SD Biosensor COVID-19 Ag test) (n = 4). (**g**) Amplification curves for negative control (NTC) samples dissolved in the different transport medium and antigen sampling buffers (n = 3). (**h**) The sampling workflow for the SARS-CoV-2 RT-SIBA assay when using different sampling medium. The swab is directly dissolved in 1 ml of medium/buffer and 20 µl is transferred to a dilution tube containing 180 µl SIBA lysis/reaction buffer. The sample is mixed by pipetting up and down multiple times and 20 µl is transferred to the SARS-CoV-2 Egoo capsule and analysed in the Egoo instrument.

Since samples must be diluted 10-fold to reduce non-target amplification in the oropharyngeal swab we wanted to investigate whether other sampling media such as UTM and VTM can be used with the Egoo Health System. We also tested different media used for antigen quick tests. We simulated SARS-CoV-2 positive samples by spiking SARS-CoV-2 virus culture (Hong Kong/VM2000i06i/2020 or (USA-WA1/2020)) into PBS, UTM (Copan), VTM (Nest Biotechnology), VTM (Mole Bioscience), Gensure antigen buffer, Acro antigen buffer, Biosynex antigen buffer or SD Biosensor antigen buffer (1:10). The simulated samples were further diluted 10-fold in SIBA lysis/reaction buffer before the samples were analysed with the SARS-CoV-2 Egoo capsule on the Egoo instrument (Fig. 4). The SARS-CoV-2 RT-SIBA assay was not influenced by the presence of UTM (Copan), VTM (Nest Biotechnology), VTM (Mole Bioscience), and all dilutions of the virus culture (Fig. 4a,b) were detected except the SARS-CoV-2 negative samples (Fig. 4g). In addition, we observed no difference between SARS-CoV-2 positive samples dissolved in PBS and Acro antigen buffer, Biosynex antigen buffer or SD Biosensor antigen buffer (Fig. 4d–f). However, SARS-CoV-2 positive samples dissolved in Gensure antigen buffer were inhibited (Fig. 4c). These results show that the SARS-CoV-2 RT-SIBA assay is dependent on the sample collection media and only the above-mentioned medias have been validated for the SARS-CoV-2 Egoo capsule. Figure 4h shows an overview of the SARS-CoV-2 Egoo Health system when using other sampling media.

### Sample stability in SIBA lysis/reaction buffer

**Figure 5:**
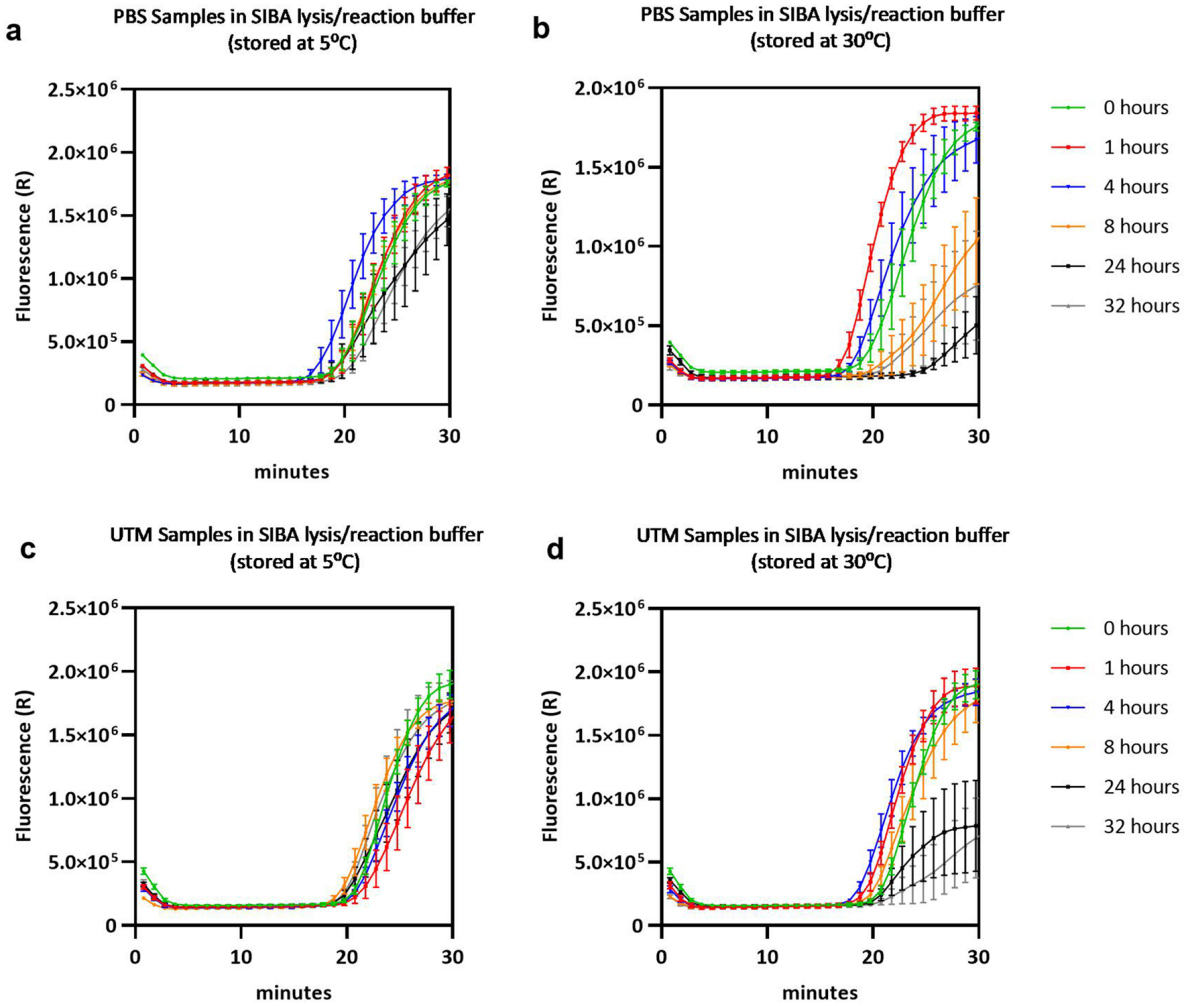
Analysis of sample stability in SIBA lysis/reaction buffer at different temperatures. (**a**,**b**) Amplification curves for SARS-CoV-2 virus simulated oropharyngeal PBS samples, 10-fold diluted in SIBA lysis/reaction buffer stored at 5 °C or 30 °C respectively for different timepoints. (**c**,**d**) Amplification curves for SARS-CoV-2 virus simulated oropharyngeal UTM samples, 10-fold diluted in SIBA lysis/reaction buffer stored at 5 °C or 30 °C respectively for different timepoints. The oropharyngeal samples were spiked with 39 viral RNA copies/µl of the heat inactivated SARS-CoV-2 (USA-WA1/2020) resulting in a finale viral concentration of 3.9 viral RNA copies/µl after dilution in SIBA lysis/reaction buffer. After storage 20 µl of the sample was transferred to the SARS-CoV-2 Egoo capsule and analysed in the Egoo instrument. The mean of five replicates with the standard error of the mean (SEM) for each timepoint.

The SARS-CoV-2 RT-SIBA reaction is dependent on the SIBA lysis/reaction buffer containing mild detergents and magnesium. The stability of SARS-CoV-2 in oropharyngeal samples when diluted 10-fold in SIBA lysis/reaction buffer was tested at different timepoints after dilution and at different temperatures. SARS-CoV-2 virus culture (USA-WA1/2020) was spiked into SARS-CoV-2 negative oropharyngeal PBS or UTM (Copan) samples resulting in a final concentration of 39 viral SARS-CoV-2 RNA copies/µl sample. The simulated SARS-CoV-2 positive samples were further diluted 10-fold in SIBA lysis/reaction buffer before the samples were analysed with the SARS-CoV-2 Egoo capsule in the Egoo instrument (Fig. 5). Samples stored at 5 °C in SIBA lysis/reaction buffer were stable up to 24 h (Fig. 5a,c). However, when stored at 30 °C the SIBA lysis/reaction buffer samples were stable up to 4 h for oropharyngeal PBS samples (Fig. 5b) and 8 h for oropharyngeal UTM samples (Fig. 5d). No difference in time to positive was observed for samples store at 5 °C compared to samples stored at 30 °C.

### Comparison to reference RT-PCR assays and platforms

The direct lysis and 10-fold dilution of the sample could potentially result in a lower clinical sensitivity of the SARS-CoV-2 RT-SIBA assay. To test this, the performance of the SARS-CoV-2 Egoo capsule on the Egoo instrument was tested on patient samples diagnosed positive or negative for SARS-CoV-2 using different RT-PCR platforms. Two of the studies were performed independently at two different Danish hospitals. In total 1154 patient samples were tested with the SARS-CoV-2 Egoo Health system and compared to either; (1) direct lysis using SIBA lysis/reagent buffer (no NA purification) followed by RT-PCR for the E-gene^33^ (Qlife COVID-19 Service Center), (2) the SARS-CoV-2 Roche Flow/MGI-BGI RT-PCR assay (Hvidovre and Amager hospitals), (3) the Cobas SARS-CoV-2 & Influenza A/B Nucleic acid test on the Cobas Liat System (Nordsjællands hospital). The comparison study showed a clinical sensitivity between 87.8 and 98.4% (Table 3). Dividing the patient samples into groups based on the ct-values from the RT-PCR analysis we find a sensitivity of the SARS-CoV-2 Egoo capsule in the Egoo instrument for samples with ct-values ≤ 35, ≤ 36, ≤ 37, ≤ 38 range from 98.4% (95% CI 94.3–99.8), 97.5% (95% Cl 93.3–99.5) to 96.5% (95% Cl 91.2–88.5) respectively for the direct lysis RT-PCR method and from 96.6% (95% Cl 94.5–98.0), 95.9% (95% Cl 93.7–97.5), 94.7% (95% Cl 92.4–96.5) to 93.8% (95% Cl 91.4–95.8) respectively for the Roche Flow/MGI-BGI RT-PCR assay. The Cobas LIAT System does not return results as ct-values and therefore the division of the samples based on ct-values can not be performed for the Cobas LIAT comparison study. These results show that a 10-fold dilution of the sample in SIBA/reaction buffer will result in a lower clinical sensitivity compared to standard RT-PCR methods, however only samples with very high ct-values will not be detected with the SARS-CoV-2 Egoo Health System.

**Table 3:** The clinical sensitivity and specificity of the SARS-CoV-2 Egoo Health system compared to different RT-PCR platforms (n = 1154).

NA and RT-PCR platform	Material	CT	P	N	TP	TN	FP	FN	SE (%)	95% Cl (%)	SP (%)	95% Cl (%)
Direct lysis and RT-PCR^a^	OP in PBS	≤ 45	133	98	127	95	3	6	95.5	90.4–98.3	96.9–100*	91.3–99.4
		≤ 38	133	98	127	95	3	6	95.5	90.4–98.3		
		≤ 37	131	98	127	95	3	4	96.9	92.4–99.3		
		≤ 36	130	98	127	95	3	3	97.7	93.4–99.5		
		≤ 35	129	98	127	95	3	2	98.5	94.5–99.8		
Roche Flow/MGI-BGI RT-PCR^b^	OP in UTM	≤ 45	525	175	482	169	6	43	91.8	89.1–94.0	96.6–100.0*	92.7–98.7
		≤ 38	504	175	473	169	6	31	93.9	91.4–95.8		
		≤ 37	494	175	468	169	6	26	94.7	92.4–96.5		
		≤ 36	482	175	462	169	6	20	95.9	93.7–97.5		
		≤ 35	466	175	450	169	6	16	96.6	94.5–98.0		
Cobas LIAT System^c^	OP in UTM	N/A	115	109	100	107	2	15	87.0	79.4–92.5	98.2	93.5–99.8

NA, nucleic acids; P, positive; N, negative; TP, true positive; TN, true negative; FP, false positive; FN, false negative; SE, sensitivity; SP, specificity; CI, confidence interval; OP, oropharyngeal swab; PBS, phosphate-buffered saline, UTM, universal transport medium.

^a^Diagnosed with direct lysis using SIBA lysis/reaction buffer and the RT-PCR for the E-gene^33^.

^b^Diagnose with the SARS-CoV-2 Roche Flow/MGI-BGI RT-PCR.

^c^Diagnosed with the Cobas SARS-CoV-2 & Influenza A/B NAAT test.

*Based on evaluation of the curves and not the Clinical app.

The specificity of the SARS-CoV-2 RT-SIBA assay in the Egoo instrument ranged from 96.6 to 98.2% (Table 3) showing that the 10-fold dilution of the sample in SIBA lysis/reaction buffer significantly reduces the non-target amplification of the SYBR based assay. The results were obtained using the Clinical app, but if we evaluate the results by the slope of the amplification curves of the true positives and false positives, the big comparison study (n = 1154) showed a clear difference in the slope of the curves (Supplementary Fig. S5) which could indicate that adjustments to the current algorithm could increase the specificity of the SARS-CoV-2 RT-SIBA assay on the Egoo instrument to 98.2–100%.

## Discussion

Here, we present for the first time a very compact instrument called the Egoo Health System, which has been developed for home-use monitoring of biochemical markers. The Egoo Health System is simple to use and can be used in private homes, primary care clinics, nursing homes, and workplaces without the need for specialized laboratory staff.

The Egoo Health System uses specialized Egoo capsules that are sealed in the Egoo instrument with a piston mechanism and a plunger. Once the plunger has sealed the capsule tight, the reaction begins which eliminates the risk of amplicon contamination. The amplification steps in NAAT tests are extreme, resulting in billions of copies of the target of interest. This amplification step requires a closed system to avoid amplicon contamination and the detection of false positives. Opening a tube after an amplification to use it on e.g. a lateral flow stick^19,36^ is possible to do in a specialised laboratory but is not possible to do outside a laboratory in the current form without a high risk of contaminating the surroundings and the following patient samples. Recently other closed systems have been developed such as the LuciraCOVID-19 All-in-One single-use Test kit (https://www.lucirahealth.com/) and the single-use COVID-19 test from Visby medical (https://www.visbymedical.com/) which are all-in-one single use NAAT tests that eliminates the need for opening the tube after amplification. In contrast to these single-use molecular test kits, the Egoo Health System can be used unlimited times and only requires replacing the Egoo assay capsule after use. Therefore, the Egoo Health System can be used for many subsequent assays, including tests for other respiratory viruses and biochemical markers such C-reactive protein (CRP) and Phenylalanine (PHE) (https://www.egoo.health).

Due to limiting heating capacity of the small Egoo instrument, we developed an isothermal SARS-CoV-2 assay based on RT-SIBA^28,29^ and SYBR green detection. The SARS-CoV-2 RT-SIBA assay is performed at 44 °C and can be used in both the Egoo instrument and in high-throughput format using standard PCR instruments. The gold standard RT-PCR test is dependent on the heating and cooling of the sample for the amplification reaction to occur. Heating and cooling require specialized equipment and so far, the most common PCR instruments have been too big and heavy to handle outside the laboratory. Recently, the CovidNudge portable RT-PCR platform^27^ and the single-use RT-PCR device from Visby medical^26^ were developed which opens the possibility to perform the gold standard RT-PCR outside the laboratory. The Egoo instrument uses similar fluorescence optics as a PCR instrument and as such we observe a similar performance of the SARS-CoV-2 RT-SIBA assay in the two instruments with an analytical sensitivity of 25 viral RNA copies per reaction.

For methodological simplicity, we developed an extraction-free SARS-CoV-2 RT-SIBA assay that uses a specialized SIBA lysis/reaction buffer containing mild detergents. During the COVID-19 pandemic, NA extraction has proved not only to be time-consuming, and has caused bottlenecks due to lack/shortage of consumables. Therefore, many laboratories have been forced to look for alternative methods to NA extraction such as direct use of the crude sample using either heat or detergents for inactivation and lysis of the virus. This has proven to be almost as sensitive and specific as the gold standard purification methods^5–7,9,37^ and after optimization, we ended with a simplified sampling workflow for the SARS-CoV-2 Egoo capsule that can be used with the Egoo instrument.

When performing the SARS-CoV-2 RT-SIBA assay in a PCR instrument a melting curve analysis can be performed to test the specificity of the assay. However, this is not possible in the Egoo instrument and therefore sample dilution must be performed to reduce non-target amplification, e.g., by adding a high sampling volume (10 ml) to the collection tube or diluting the sample 10-fold. Studies have shown that direct use of nasopharyngeal or oropharyngeal samples dissolved in PBS, Saline, and UTM without NA extraction can inhibit direct RT-PCR^9,37^ and dilution of samples (or reducing the sample input volume into the RT-PCR reaction) reduced the inhibitory effect^37^. The 10-fold dilution of the sample before analysis in the Egoo instrument will therefore not only eliminate non-target amplification but may also eliminate inhibitors that might otherwise influence the SARS-CoV-2 RT-SIBA reaction. Another advantage of the dilution workflow is that the SARS-CoV-2 RT-SIBA assay is compatible with several different VTMs, UTMs and antigen buffer systems. We show that SARS-CoV-2 Egoo Health System is compatible with several of the widely used antigen buffers systems and therefore can be used as a confirmatory NAAT test of patients tested positive or negative with a rapid antigen test^4^.

The SARS-CoV-2 Egoo Health System showed a sensitivity between 87.0 and 94.7% dependent on the reference NAAT test used. We obtained the lowest sensitivity of 87.0% (100/115) when the assay was compared to the Cobas SARS-CoV-2 & Influenza A/B NAAT test on the Cobas Liat System. The main reason for this difference is the sample input volume into the system. For the Corbas Liat system 200 µl sample is loaded directly into the assay cartridge, whereas for the Egoo system 20 µl sample is 10-fold diluted before 20 µl of the diluted sample is loaded into the SARS-CoV-2 Egoo capsule, meaning that there is a 100-fold difference in sample input between the two systems. Compared to the Roche Flow/MGI-BGI RT-PCR reference method which include a NA purification, an up concentration (180–33 µl) of the RNA and detection of two targets, we obtained a sensitivity of 91.8% (482/525) with the SARS-CoV-2 Egoo Health System. For samples with ct-values below 35 the Egoo system achieved a sensitivity of 96.6% (450/466) meaning that samples with low viral load (high ct-values) could potentially not be detected with the Egoo Health System.

The clinical performance of the SARS-CoV-2 Egoo Health system is similar to the clinical performance of the COVID-19 test on the Cue Health Monitoring system^25^. The Cue Health Monitoring System (Cue Cartridge Reader) have recently developed a COVID-19 Test Cartridge which was evaluated on 292 symptomatic and asymptomatic patients^25^. They showed a positive percent agreement (PPA) of 91.7% (22/24) and a negative percent agreement (NPA) of 98.4% (239/243) compared to a reference NAAT test using standard nasopharyngeal swab^25^ which is comparable to our sensitivity and specificity of 91.8% and 98.2% respectively when testing 1154 oropharyngeal swabs.

The Egoo Health System and SARS-CoV-2 RT-SIBA assay presented here has recently been CE-marked for professional use (Qlife), and we are currently trying to develop a multiplex probe based SARS-CoV-2 RT-SIBA assay containing the human RNaseP as an internal control^38,39^ that can be used directly on anterior nasal swabs.

## Supplementary Information

Supplementary Information.

## Data Availability

Data sharing not applicable to this article as no datasets were generated.
