# Supplementary material for: SARS-CoV-2 detection using reverse transcription strand invasion based amplification and a portable compact size instrument
Source: Sci Rep. 2021 Nov 15;11:22214. doi: 10.1038/s41598-021-01744-y (PMC8593107; doi:10.1038/s41598-021-01744-y)
Supplement: Supplementary file 1 — Supplementary Information. [file 41598_2021_1744_MOESM1_ESM.docx]

**SARS-CoV-2 detection using Reverse Transcription Strand Invasion Based Amplification and a portable compact size instrument.**

Maiken W. Rosenstierne^1,2*^, Shreya Joshi^1^, E. Thomas Danielsen^1^, Helen Webb^1^, Dac Mui Luong^1^, Julie Bjerring^1^, Julie Hindkær^1^, Lærke Jørgensen^1^, Julie Blauenfeldt^1^, Ask Bojesen^1^, Flemming Holck^1^, Johnny Weber Lau^1^, Lars Bangsgaard^1^, Jakob Broberg Lind^1^, Mette Bjergaard Dragheim^1^, Mikkel Rohde Jacobsen^1^, Robert Elkjær^1^, Steven Clauwaert^1^, Kristina Christensen^1^, Charlotta Polacek^2^, Anders Fomsgaard^2^, Tuomas Ojalehto^3^, Antti Tullila^3^, Mirko Brummer^3^, Claus Juel Jensen^4^, Frederikke Holm Jensen^5^, Uffe Vest Schneider^5^, Jan Gorm Lisby^5^, Rikke Lind Jørgensen^5^, Thomas Warthoe^1^, Ebbe Finding^1^ and Peter Warthoe^1^

^1^ Qlife, Borupvang 3, 2750 Ballerup & Symbion, Fruebjergvej 3, 2100 Copenhagen, Denmark

^2^ Department of Virus and Microbiological Special diagnostics, Statens Serum Institut, Artillerivej 5, 2300 Copenhagen, Denmark

^3^ Aidian Oy, Espoo, Finland

^4^ Klinisk Biokemisk Afdeling, Nordsjællands Hospital, Dyrehavevej 29, 3400 Hillerød, Denmark

^5^ Amager og Hvidovre Hospital, Klinisk Mikrobiologisk Afdeling, afsnit 445, Kettegård Allé 30, 2650 Hvidovre, Denmark

*Corresponding author; Maiken W. Rosenstierne, Mwr@egoo.health

**Supplementary information**

**Supplementary Table S1: Egoo device fact box**

| Dimensions | Metric: W66 x H107 x D94 mm  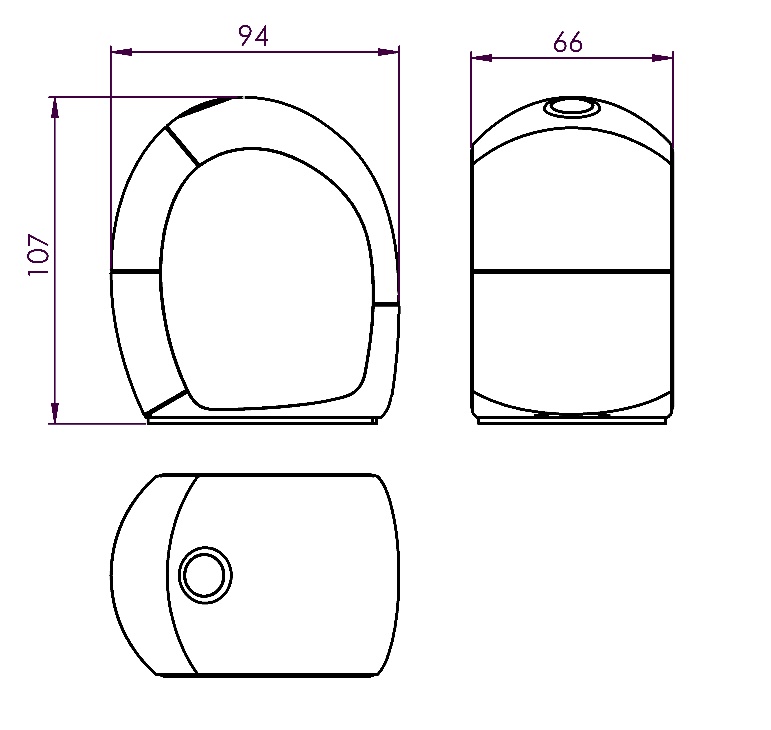Imperial: W2,60 x H4,21 x D3,70 “ |
| --- | --- |
| Weight | 470 g (1.04 lbs) |
| Detection principle | Fluorescence, absorbance and turbidimetry |
| Detection unit | An optical MOEM consisting of a dual optical unit for simultaneously measuring fluorescence and absorbance. The optical unit is integrated into a micro heating and vortex mixer unit for heating and mixing. |
| Mechanics | Heating unit (50°C (122°F))  Mixing unit (max. 3000 rpm.)  Drawer mechanism for placing the assay-specific capsule  Piston/plunger mechanism for activating the injection chambers on board the assay capsules |
| Electronics | A dual-processor system with integrated Wi-Fi radio.  A very low noise AFE with 24-bit Δ∑ ADC for optimal results.  Touch capacitive capability and simple user interface.  On board communication via I2C, I2S and SPI protocols |
| Communication | Wi-Fi 2,4GHz |
| Power supply | Medical AC/DC Adapter, Input: 100-240V 50/60Hz 0.5A, Output: 24VDC 0.75A |

**Supplementary Table S2: Egoo Sars-CoV-2 capsule fact box**

| Dimensions | Metric: W24 x H28 x D36 mm  Imperial: W0,95 x H1,10 x D1,42 “  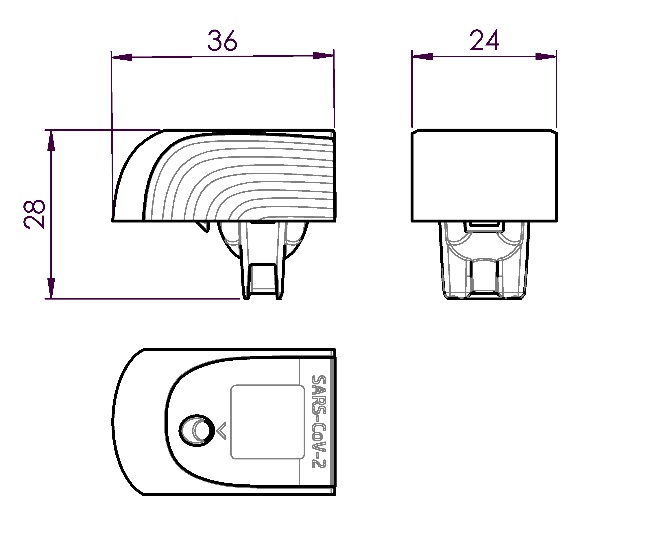 |
| --- | --- |
| SARS-CoV-2 RT-SIBA capsule | Closed assay container for adding the oropharyngeal lysate |
| Top part of the capsule | For guiding the injecting of the test sample test using a mini fixed pipet |
| Upper part of the capsule | The injection chambers containing the various assay reagents |
| Lower capsule part | Main cuvette where the SARS-CoV-2 RT-SIBA reaction is performed  Each injection chamber can realise the assay reagents into this main cuvette |
| Time to result | 30 min. |
| Disposal | After end run, the assay capsule is disposed as biological waste |

**Supplementary fig. S1**

**
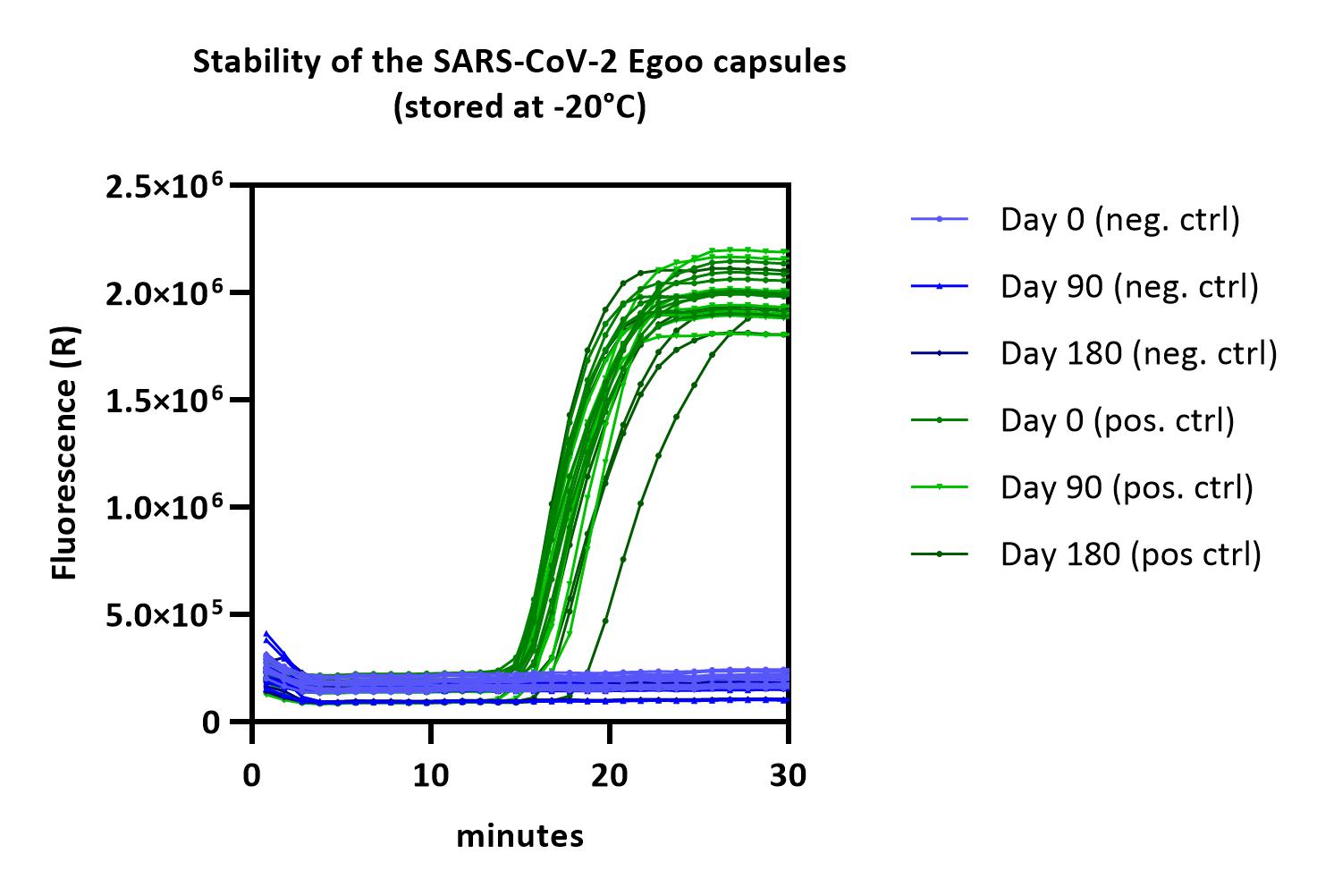
**

**Fig. S1. Shelf-life stability of the SARS-CoV-2 Egoo capsules stored at -20°C.** Amplification curves for CE-IVD SARS-CoV-2 positive and negative RNA RUN controls (BioRAD) analysed with the SARS-CoV-2 Egoo capsules stored at -20 °C for up to 180 days. The SARS-CoV-2 positive (2,000 RNA copies/µl) and negative RUN controls were 10-fold diluted in PBS before 20 µl of the dilution was transferred to 180 µl of SARS-CoV-2 lysis/reaction buffer resulting in a final concentration of 20 RNA copies/µl. The sample was mixed by pipetting and down for 5-10 times before 20 µl of the sample was transferred to the SARS-CoV-2 Egoo capsule containing 140 µl of SARS-CoV-2 RT-SIBA mastermix. The capsule was then inserted into the Egoo Instrument and analyzed for 30 minutes. The positive SARS-CoV-2 RUN controls are shown in green, and the negative SARS-CoV-2 RUN controls are shown in blue (n=5 per timepoint).

**Supplementary fig. S2:**

**
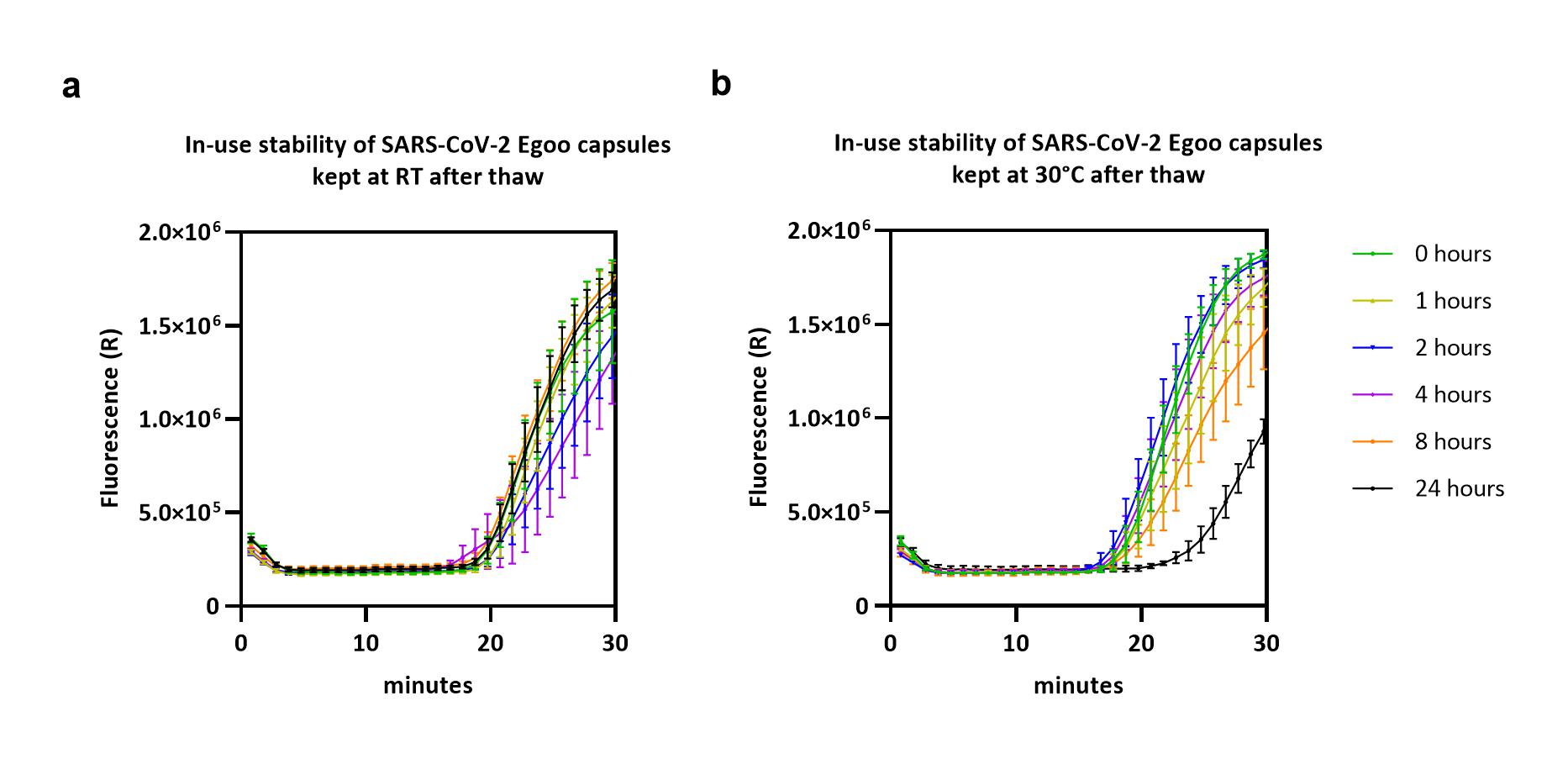
**

**Fig. S2. In-use stability of the SARS-CoV-2 Egoo capsule after thawing.** The SARS-CoV-2 Egoo capsule can be stored at -20°C until use. Upon use the capsule was thawed before applying 20 µl of sample (10-fold diluted in SIBA lysis/reaction buffer). **a)** Amplification curves for simulated SARS-CoV-2 virus positive oropharyngeal PBS samples analysed in SARS-CoV-2 Egoo capsules that have been kept at RT for different hours after thawing. **b**) Amplification curves for simulated SARS-CoV-2 virus positive oropharyngeal PBS samples analysed in SARS-CoV-2 Egoo capsules that have been kept at 30°C for different hours after thawing. After keeping the capsule at RT or 30°C, 20µl of the sample was transferred to the SARS-CoV-2 Egoo capsule and analysed in the Egoo instrument. The mean of five replicates with the standard error of the mean (SEM) for each timepoint. The different timepoints are shown in different colors.

**Supplementary fig. S3:**

**
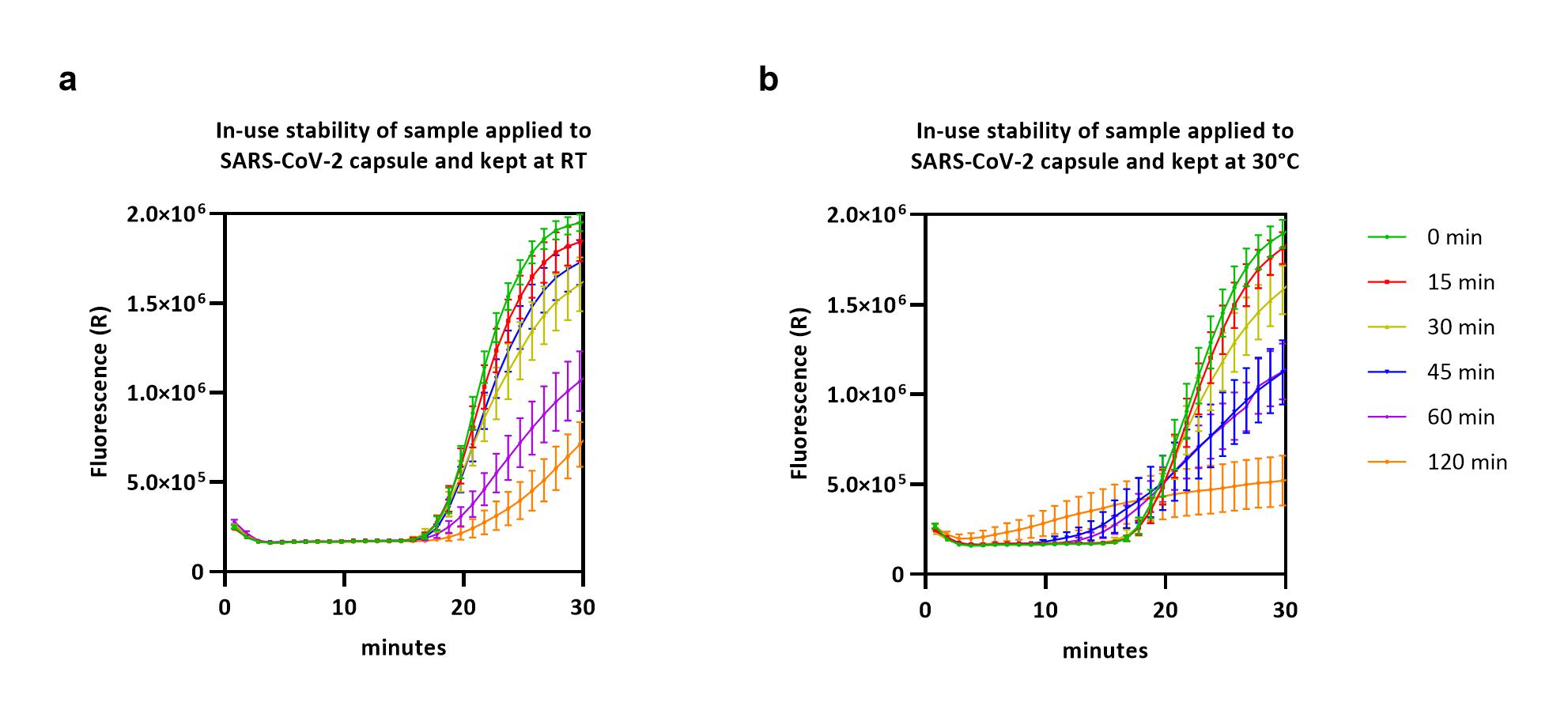
**

**Fig. S3. In-use stability of sample applied to SARS-CoV-2 Egoo capsule.** After thawing the SARS-CoV-2 Egoo capsule at RT, 20 µl of sample (diluted 10-fold in SIBA lysis/reaction buffer) was applied to the Egoo capsule and kept at RT or 30 °C for several minutes before being analyzed in the Egoo instrument. **a)** Amplification curves for simulated SARS-CoV-2 virus positive oropharyngeal PBS samples analysed in SARS-CoV-2 Egoo capsules that have been kept at RT for several minutes after applying the sample. **b**) Amplification curves for simulated SARS-CoV-2 virus positive oropharyngeal PBS samples analysed in SARS-CoV-2 Egoo capsules that have been kept at 30°C for several minutes after applying the sample. The loaded SARS-CoV-2 Egoo capsules were transferred to the Egoo instrument for analysis. The mean of five replicates with the standard error of the mean (SEM) for each timepoint. The different timepoints are shown in different colors.

**Supplementary Table S3*: In Silico* analysis of SNPs in the SARS-CoV-2 RT-SIBA assay**

| **Country** | **Number of sequences analyzed for SNPs** | **Number of sequences with SNPs** | **WT (%)** | **SNPs (%)** |
| --- | --- | --- | --- | --- |
| Argentina | 1440 | 1 | 99.93 | 0.07 |
| Australia | 12930 | 3 | 99.98 | 0.02 |
| Austria | 2144 | 5 | 99.77 | 0.23 |
| Bangladesh | 1016 | 1 | 99.90 | 0.10 |
| Belgium | 7730 | 15 | 99.81 | 0.19 |
| Brazil | 8489 | 55 | 99.35 | 0.65 |
| Canada | 19941 | 174 | 99.13 | 0.87 |
| Chile | 1713 | 6 | 99.65 | 0.35 |
| Colombia | 353 | 1 | 99.72 | 0.28 |
| Congo | 237 | 2 | 99.16 | 0.84 |
| Croatia | 669 | 4 | 99.40 | 0.60 |
| Denmark | 46799 | 108 | 99.77 | 0.23 |
| Egypt | 693 | 2 | 99.71 | 0.29 |
| England | 131786 | 319 | 99.76 | 0.24 |
| Estonia | 577 | 2 | 99.65 | 0.35 |
| Finland | 2494 | 1 | 99.96 | 0.04 |
| France | 10538 | 17 | 99.84 | 0.16 |
| Germany | 29353 | 52 | 99.82 | 0.18 |
| Iceland | 4255 | 2 | 99.95 | 0.05 |
| India | 9402 | 16 | 99.83 | 0.17 |
| Indonesia | 1498 | 1 | 99.93 | 0.07 |
| Ireland | 2792 | 2 | 99.93 | 0.07 |
| Israel | 2590 | 6 | 99.77 | 0.23 |
| Italy | 7471 | 31 | 99.59 | 0.41 |
| Japan | 33554 | 45 | 99.87 | 0.13 |
| Jordan | 542 | 3 | 99.45 | 0.55 |
| Latvia | 2021 | 7 | 99.65 | 0.35 |
| Lithuania | 2703 | 1 | 99.96 | 0.04 |
| Luxembourg | 3963 | 5 | 99.87 | 0.13 |
| Malaysia | 526 | 3 | 99.43 | 0.57 |
| Mexico | 6238 | 16 | 99.74 | 0.26 |
| Netherlands | 10876 | 18 | 99.83 | 0.17 |
| Nigeria | 304 | 1 | 99.67 | 0.33 |
| Norway | 3662 | 10 | 99.73 | 0.27 |
| Panama | 326 | 1 | 99.69 | 0.31 |
| Philippines | 1545 | 11 | 99.29 | 0.71 |
| Poland | 2124 | 11 | 99.48 | 0.52 |
| Portugal | 3557 | 66 | 98.14 | 1.86 |
| Qatar | 629 | 3 | 99.52 | 0.48 |
| Romania | 343 | 3 | 99.13 | 0.87 |
| Russia | 2368 | 12 | 99.49 | 0.51 |
| Senegal | 217 | 1 | 99.54 | 0.46 |
| Serbia | 230 | 2 | 99.13 | 0.87 |
| Singapore | 1149 | 12 | 98.96 | 1.04 |
| Slovakia | 1486 | 2 | 99.87 | 0.13 |
| Slovenia | 4285 | 5 | 99.88 | 0.12 |
| Spain | 12780 | 61 | 99.52 | 0.48 |
| Sweden | 13619 | 31 | 99.77 | 0.23 |
| Switzerland | 17808 | 51 | 99.71 | 0.29 |
| Thailand | 514 | 4 | 99.22 | 0.78 |
| Togo | 63 | 1 | 98.41 | 1.59 |
| Tunisia | 91 | 1 | 98.90 | 1.10 |
| Turkey | 3523 | 3 | 99.91 | 0.09 |
| Uganda | 323 | 6 | 98.14 | 1.86 |
| USA | 238745 | 1384 | 99.42 | 0.58 |
| **Total** | **699,737** | **2623** | **99.63** | **0.37** |

SNPs; Single nucleotide polymorphisms, WT; wild type

*Based on sequences uploaded to GISAID as of December 26^th^ 2019 to May 22^nd^ 2021.

**Supplementary Fig. S4**

**
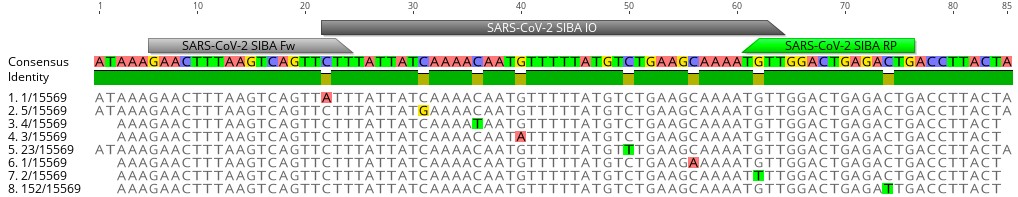
**

**Fig. S4. A representative description of the different SNPs observed in the SARS-CoV-2 RT-SIBA assay.** Different SNPs in the binding region of the primers and invasion oligo are shown for 15.569 Danish SARS-CoV-2 isolates uploaded to GISAID from January 2021 to May 2021. The number of each SNP variant is shown on each line. The most predominant SNP with 152 out of 15.569 isolates is located at the 5´end of the reverse primer. The rest of the SNPs are randomly scattered across the target region confirming that the RdRp target region is very conserved. The alignments were performed with Geneious 2021.1.1 Website: [www.geneious.com](http://www.geneious.com).

**Supplementary Table S4: Analytical specificity of the SARS-CoV-2 Egoo capsules**

| Organism | Concentration | Number of positive on the Egoo instrument |
| --- | --- | --- |
| Epstein-Barr Virus (EBV) | 2.7 x 10^8^ copies/ml | 0/10 |
| Parainfluenza Virus Type 1 (PIV-1) | 9.1 x 10^8^ copies/ml | 0/10 |
| Adenovirus Type 05 (ADV5) | 4.1 x 10^7^ TCID_50_/ml | 0/10 |
| Respiratory Syncytial Virus Type A (RSV) | 5.0 x 10^5^ TCID_50_/ml | 0/10 |
| Influenza B (Yamagata/16/88) | 2.5 x 10^5^ TCID_50_/ml | 0/10 |
| Influenza A H1N1pdm (NY/02/09) | 3.8 x 10^6^ TCID_50_/ml | 0/10 |
| Human Coronavirus 229E | 1.4 x 10^5^ TCID_50_/ml | 0/10 |
| Human Coronavirus NL63 | 4.7 x 10^4^ TCID_50_/ml | 0/10 |
| Human Metapneumovirus 3 Type B1 (hMPV) | 3.9 x 10^4^ TCID_50_/ml | 0/10 |
| Enterovirus Type 68 | 5.0 x 10^5^ TCID_50_/ml | 0/10 |
| Bordetella pertussis | 2.5 x 10^10^ genomes/ml | 0/10 |
| Candida albicans | 4.3 x 10^5^ genomes/ml | 0/10 |
| Chlamydia trachomatis | 1.7 x 10^6^ genomes/ml | 0/10 |
| Corynebacterium diphtheriae | 2.0 x 10^8^ genomes/ml | 0/10 |
| Escherichia coli | 1.5 x10^10^ genomes/ml | 0/10 |
| Haemophilius influenzae | 2.7 x 10^9^ genomes/ml | 0/10 |
| Legionella pneumophila | 1.7 x 10^10^ genomes/ml | 0/10 |
| Moraxella osloensis | 4.2 x 10^8^ genomes/ml | 0/10 |
| Mycoplasma pneumoniae | 2.9 x 10^8^ genomes/ml | 0/10 |
| Neisseria meningitidis | 5.3 x 10^8^ genomes/ml | 0/10 |
| Pseudomonas aeruginosa | 1.4 x 10^10^ genomes/ml | 0/10 |
| Staphylococcus epidermis | 2.5 x 10^9^ genomes/ml | 0/10 |
| Streptococcus pneumoniae | 7.2 x10^9^ genomes/ml | 0/10 |
| Streptococcus pyogenes | 6.0 x 10^9^ genomes/ml | 0/10 |
| Streptococcus salivarius | 3.8 x 10^7^ CFU/ml | 0/10 |
| SARS-CoV-2 Positive Run Control* | 2.0 x 10^5^ copies/ml | 10/10 |

***** CE-IVD SARS-CoV-2 positive RNA RUN control (BioRAD)

**Supplementary Fig. S5**

**
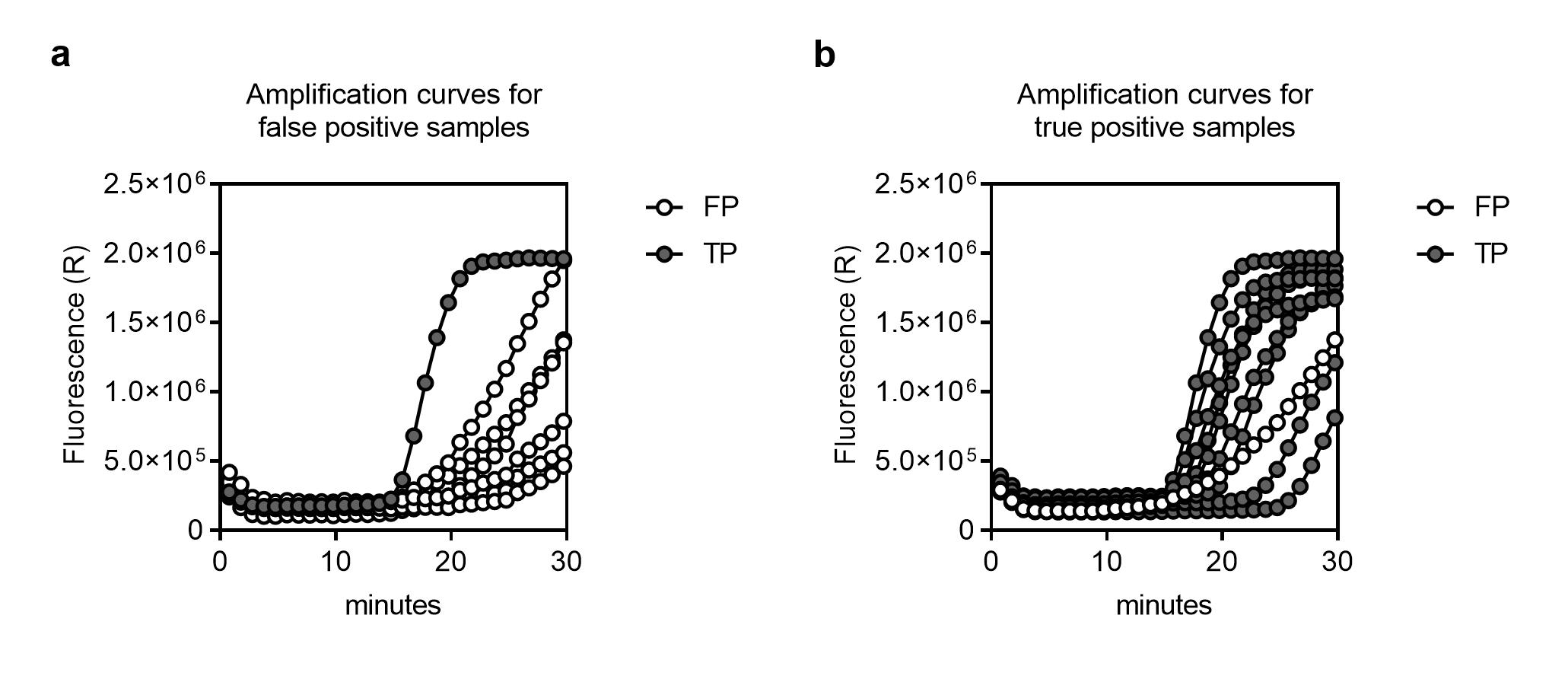
**

**Fig. S5. Amplification curves for false positive samples (n=6) and true positive samples (n=9).** **a**) Amplification curves for false positive oropharyngeal swabs analysed with the SARS-CoV-2 Egoo capsule on the Egoo system. **b**) Amplification curves for true positive oropharyngeal swabs analysed with the SARS-CoV-2 Egoo capsule on the Egoo system. The false positive (FP) amplification curves are shown in white, and the true positive (TP) amplification curves are shown in dark gray.
